# Cardiac MRI improves identification of etiology of ischemic stroke

**DOI:** 10.1186/1532-429X-14-S1-P79

**Published:** 2012-02-01

**Authors:** Alex Baher, Ashkan Mowla, John J Volpi, Dipan J Shah

**Affiliations:** 1 Internal Medicine, Methodist Hospital, Houston, TX, USA; 2 Methodist Neurological Institute, Methodist Hospital, Houston, TX, USA; 3 Methodist DeBakey Heart & Vascular Center, Methodist Hospital, Houston, TX, USA

## Background

Stroke is the third leading cause of death in the United States. Ischemic etiologies account for the vast majority of all cases of stroke from which about 20-25% have a cardiogenic cause. Transthoracic echocardiography (TTE) is widely used as the initial test for evaluating patients with a suspected cardioembolic of stroke while additional studies are often required to further detect a cardiac source in patients with unremarkable TTE. While transesophageal echocardiography (TEE) has provided clinicians with invaluable information in these cases, most physicians find it cumbersome at best and patients it extremely uncomfortable. CMR is a novel imaging modality that is increasingly being used in assessing patients with cardiovascular disease. The non-invasive nature of this test makes it an attractive option for evaluating strokes for which a TTE is non-diagnostic. In this study, we assessed the feasibility of using CMR as an imaging modality additional to TTE for detection of cardioembolic strokes.

## Methods

CMR was performed on 54 patients (25 men), age of 64.5± 16.3 years, who presented between August 2009 and June 2011 to our hospital with an acute stroke detected by diffusion weighted imaging brain MRI. In addition to the CMR all patients received a "routine" stroke workup which included a TTE, MRA of head and neck, carotid duplex, and laboratory workup. Patients without an obvious stroke etiology underwent additional studies such as transcranial duplex with or without a bubble study, and/or a cerebral angiogram. We divided patients based on the TOAST criteria for classification of ischemic stroke into atherothrombotic, cardioembolic, lacunar, other causes, and cryptogenic subtypes based on routine workup. We then assessed whether CMR could help to further identify a cardiac cause in patients with cryptogenic stroke.

## Results

Table 1 summarizes our results. Based on initial assessment we found 15 patients (27.8%) with cryptogenic stroke. In 10 of these patients, CMR detected an underlying cardiac cause (Table 2). Overall CMR findings reduced the rate of cryptogenic stroke by 66.7% in our study population. From the patients that were classified as cardioembolic, there were 2 with TTE finding of severe annular calcification of the mitral valve, a relative risk factor for cardiogenic stroke. In one of these patients, CMR detected diffuse atherosclerosis of the ascending aorta and in the other, CMR detected transmural infarction of the septum, both which were undetected by TTE.

**Table 1:** Incidence of stroke subtypes with and without CMR

Stroke etiology	Routine workup	Routine workup + CMR	P*
Cardioembolic	16 (29.6%)	26 (48.1%)	0.004
Artherothrombotic	16 (29.6%)	16 (29.6%)	1.00
Lacunar	2 (3.7%)	2 (3.7%)	1.00
Other	5 (9.3%)	5 (9.3%)	1.00
Cryptogenic	15 (27.8%)	5 (9.3%)	0.004

* Using McNemar’s test.

**Table 2:** Cardiac findings in patients who were reclassified based on CMR results

Patient	CMR findings
1	Non-CAD pattern scarring
2	Non-CAD pattern scarring
3	CAD pattern scarring
4	Ascending aortic aneurysm
5	a) Non-CAD pattern scarring b) Aortitis
6	CAD pattern scarring
7	CAD pattern scarring
8	Non-CAD pattern scarring
9	a) CAD pattern scarring b) B) Intra-atrial septal aneurysm
10	Non-CAD pattern scarring

## Conclusions

CMR is an effective non-invasive modality in identifying cardioembolic etiologies in patients with stroke. Further studies to compare the effect of CMR guided management changes on outcomes are warranted.

## Funding

None.

